# Prevalence of Human Papilloma Virus in Tunisia

**DOI:** 10.1186/1471-2334-14-S2-P95

**Published:** 2014-05-23

**Authors:** Ali Mrabet, MT Khoufi, C Tounsi, R Dhaoui, M Chibani, N Doss

**Affiliations:** 1 Tunisian Military Health General Directorate, Tunis, Tunisia

## Introduction

Cervical cancer is still a major health problem in Tunisia and over the world. HPV infection may induce malignant transformation of lesions, especially in case of colonization by oncogenic HPV genotypes. Early detection of viral genome by molecular biology in women with normal cytology in cervical screening test (CST) can be a part of a screening policy to improve cancer prevention. Our aim was to evaluate HPV prevalence and genotype distribution in routine CST.

## Methods

It was a prospective study conducted over a six month period (2012, January to June). 665 women were screened by an examination of the cervix, a cervical screening test and a detection of the HPV DNA by PCR. This detection of the high oncogenic risk HPV was made from the cytobrush that was used for the spreading of the cervix cells.

## Results

The mean age was 41.5 ±9.1 years (20-71 y). The mean age of the marriage was 24.8±5 years (16-50 y). The age of the first pregnancy was 24.8±5.7 years (14-48 y). Six percent reported history of genital lesion in their partner. 37% used or are still using Intra Uterine Device and 48% used or are still using contraceptive pills. 58% had never had a routine CST and 27% had one CST. 65% of the women reported a history of vaginal discharge, 25% a genital ulceration and 4.5% condylomas. The cervix examination showed 51% of normal cervix, without any clinical macroscopic anomaly. 41% had a cervix inflammation, 18% had leucorrhea, 7% had polypus and 3% had condylomas. HPV prevalence was 3%.

## Conclusion

Our results showed lower HPV prevalence, probably due to later onset of sexual practice and fewer sexual partners.

